# Seasonal Decline in Lung Function Among Residents of Northern Thailand: A Longitudinal Study Before and After the Haze Period

**DOI:** 10.3390/jcm15103791

**Published:** 2026-05-14

**Authors:** Anurak Wongta, Kriangkrai Chawansuntati, Supansa Pata, Woottichai Khamduang, Supachai Yodkeeree, Surat Hongsibsong

**Affiliations:** 1 School of Health Science Research, Research Institute for Health Sciences, Chiang Mai University, Chiang Mai 50200, Thailand; anurak.wongta@cmu.ac.th; 2 Research Institute for Health Sciences, Chiang Mai University, Chiang Mai 50200, Thailand; kriangkrai.ch@cmu.ac.th; 3 Environmental, Occupational, and NCD Center of Excellent, Research Institute for Health Sciences, Chiang Mai University, Chiang Mai 50200, Thailand; 4 Department of Medical Technology, Faculty of Associate Medical Science, Chiang Mai University, Chiang Mai 50200, Thailand; supansa.pata@cmu.ac.th (S.P.); woottichai.k@cmu.ac.th (W.K.); 5 Department of Biochemistry, Faculty of Medicine, Chiang Mai University, Chiang Mai 50200, Thailand; supachai.y@cmu.ac.th

**Keywords:** PM_2.5_, seasonal haze, spirometry, lung function, FEV_1_/FVC ratio, early functional changes, longitudinal study, Thailand

## Abstract

**Background**: Seasonal haze from biomass burning in northern Thailand frequently elevates PM_2.5_ above national and WHO standards, while evidence on short-term lung function changes in exposed populations remains limited. **Method:** This longitudinal study evaluated changes in lung function in 130 adults before and after the haze season from December 2023 to May 2024 in San Pa Thong District, Chiang Mai Province. Spirometry was conducted following ATS/ERS standards. Demographic and health-risk data were gathered using validated Thai questionnaires addressing diabetes, cardiovascular, and chronic obstructive pulmonary disease risk categories. **Results:** A significant reduction in the FEV_1_/FVC ratio was observed, from 85.3% to 82.4% (*p* = 0.001) after the haze period, while the proportion of participants with FEV_1_/FVC < 70% increased from 2.3% to 6.9% (*p* = 0.031). No statistically significant independent predictors were identified in multivariable analysis. Greater reductions were observed in older adults, low-income individuals, and those at moderate to high diabetes risk. **Conclusions:** These findings suggest modest changes in spirometry parameters during the seasonal haze period. The observed reduction in FEV_1_/FVC should be interpreted cautiously and may reflect early functional variation rather than confirmed airway obstruction. Although the magnitude of change was relatively small, the findings highlight the potential value of ongoing respiratory monitoring and early detection strategies in haze-affected regions.

## 1. Introduction

In low- and middle-income countries, particularly in Southeast Asia, air pollution represents a critical public health challenge, exacerbated by seasonal haze and the combustion of biomass, which causes increased levels of fine particulate matter (PM_2.5_). The northern area of Thailand experiences ongoing haze issues throughout every dry season, mostly resulting from the burning of farming by-products and forest fires. During these periods, ambient PM_2.5_ levels often surpass national standards and the World Health Organization (WHO) guidelines by several times, posing substantial environmental and health challenges [1,2]. The PM_2.5_ air quality criteria for Thailand in 2023 are set at 15 µg/m^3^ for yearly assessments and 37.5 µg/m^3^ for assessments over a 24 h timespan [3]. Nevertheless, these levels surpass WHO recommendations, underscoring the ongoing exposure challenges encountered by northern regions like Chiang Mai.

Long-term exposure to PM_2.5_ is associated with respiratory diseases such as asthma and chronic obstructive pulmonary disease (COPD). The primary mechanisms involve oxidative stress and airway inflammation. PM_2.5_ can infiltrate into the lungs and generate reactive oxygen species (ROS) that disrupt the balance of oxidative stress, leading to cellular damage and inflammation in lung tissues [4,5]. Long-term PM_2.5_ exposure is associated with reduced lung function in Asians. Previous research indicates links between higher PM_2.5_ levels and reduced lung metrics, with inflammation and PAH metabolites as potential mediators. These results underscore the urgency for enhanced air quality regulations to avert respiratory decline [6,7,8,9,10].

Recent studies in East and Southeast Asia have reported consistent evidence that long-term exposure to PM_2.5_ associated with impaired lung function. An air quality improvement was associated with forced vital capacity (FVC) and forced expiratory volume in one second (FEV_1_) recovery in young adults [11]. Long-term exposure has been associated with reduced lung growth and increased airway inflammation in children and has caused measurable declines among exposed workers [12]. Meta-analytic data confirm that each 10 µg/m^3^ increase in PM_2.5_ corresponds to decreases of about 15.6 mL in FEV_1_ and 25.3 mL in FVC [13].

The seasonal haze in northern Thailand has been significantly linked to both respiratory and cardiovascular health concerns, particularly in rural populations. Exposure to haze has been reported to correlate with increased triglyceride and HbA1c levels and decreased spirometry values, indicating metabolic alterations may contribute to variations in lung function in agricultural adults [14]. Pulmonary dysfunction is reported in approximately one-sixth of residents in high-pollution areas, with small airway disease being predominant. High amounts of PM_10_ and PM_2.5_ are associated with greater respiratory health problems, highlighting the need for community education and pollution management plans [15,16]. Seasonal increases in particulate matter have been shown to exacerbate respiratory and metabolic conditions, supported by local and regional studies [17]. Collectively, these insights emphasize the urgent need for ongoing air quality governance and focused interventions to safeguard at-risk populations in northern Thailand.

From a clinical perspective, early changes in spirometry parameters, particularly reductions in the FEV_1_/FVC ratio, may reflect early functional alterations suggestive of subclinical airway obstruction that precedes clinically apparent respiratory disease [18]. Detecting such early functional alterations in high-exposure populations may provide an opportunity for timely clinical monitoring and risk stratification.

To address this gap, the present study aimed to evaluate longitudinal changes in spirometry parameters among adults living in Chiang Mai before and after the haze season. This follow-up investigation builds upon previous baseline research on respiratory health in agricultural populations [19]. The study specifically aimed to (1) compare spirometry indices (FVC, FEV_1_, and FEV_1_/FVC) before and after the haze season, and (2) identify demographic and health factors linked to changes in FEV_1_/FVC. These insights are vital for guiding evidence-based prevention in haze-affected regions of northern Thailand and may support both public health interventions and clinical surveillance strategies.

## 2. Materials and Methods

### 2.1. Study Design and Participants

This longitudinal study involved adults in San Pa Thong District, Chiang Mai Province, Northern Thailand, an agricultural region often impacted by haze in the dry season. One hundred forty five participants were initially enrolled in the baseline study [19]. We recruited individuals aged 18 years and older who completed spirometry assessments at both points. Participants with acute respiratory infection, pregnancy, or incomplete spirometry data were excluded. A total of 130 participants were included in the study. No substantial differences in baseline characteristics were observed between participants who completed follow-up and those who were not included in the final analysis.

### 2.2. Ethics Approval and Consent to Participate

This study was approved by the Ethics Committee of the Faculty of Associated Medical Sciences, Chiang Mai University (Doc No. AMSEC-66EX-062). All participants provided written informed consent prior to participation. The study was conducted in accordance with the Declaration of Helsinki.

### 2.3. Data Collection and Timing

Two spirometry assessments were performed per participant: the initial during low PM_2.5_ levels (30 October–1 December 2023) and the subsequent following high levels (8–11 May 2024) during the seasonal haze period. Both evaluations occurred in San Pa Thong District, Chiang Mai Province, an agricultural region often impacted by seasonal haze. Regional air monitoring data from Cao et al. [20] revealed that mean monthly PM_2.5_ concentrations in San Pa Thong varied from approximately 10–15 µg/m^3^ during low-exposure months (October–December) to 40–50 µg/m^3^ during high-exposure haze season (February–April), significantly surpassing the WHO guideline of 15 µg/m^3^ by about threefold. This trend corroborated that the post-haze data collection period followed considerable exposure to elevated PM_2.5_ levels, representing cumulative or residual exposure conditions rather than peak exposure at the time of measurement. PM_2.5_ exposure was not measured at the individual level; therefore, exposure assessment in this study reflects ecological exposure based on regional monitoring data.

Spirometry was performed by trained technicians certified by the Thoracic Society of Thailand under Royal Patronage (Certificate No. 178/2566). Demographic and health-related data were obtained from a screening questionnaire that included risk assessments for diabetes mellitus (DM), cardiovascular disease (CV), and chronic obstructive pulmonary disease (COPD) [19].

### 2.4. Spirometry Procedure

Spirometry was performed using a SpiroScout device (Ganshorn Medizin Electronic GmbH, Niederlauer, Germany) following the American Thoracic Society/European Respiratory Society (ATS/ERS) standards [18]. Calibration was performed daily according to ATS/ERS 2019 guidelines. Participants were instructed to avoid smoking, caffeine, large meals, and vigorous exercise for at least two hours before the test. Participants were also asked to refrain from using short-acting bronchodilators prior to testing where applicable; however, regular medication use was not systematically recorded due to the low prevalence of diagnosed respiratory disease in this cohort. Measurements were conducted with participants seated and wearing a nose clip.

Each participant performed at least three acceptable forced expiratory maneuvers until repeatability criteria were met (difference between the two highest FEV_1_ and FVC values ≤ 150 mL). The highest acceptable values were recorded. The parameters measured included FVC, FEV_1_, and the FEV_1_/FVC ratio. Results were expressed as percent predicted values using Thai reference equations [21].

Definition of lung function decline: Lung function decline was defined as a reduction in FEV_1_/FVC (%) between the two assessments. An obstructive pattern was identified when FEV_1_/FVC < 70%, consistent with the ATS/ERS recommendations [18]. Although a fixed ratio of 70% was used for interpretability and comparability with prior studies, it may overestimate obstruction in older populations compared to the lower limit of normal (LLN), and results should be interpreted with caution in this context.

### 2.5. Statistical Analysis

All statistical analyses were conducted using IBM SPSS Statistics version 20. Continuous variables were summarized as medians with interquartile ranges (IQRs), and categorical variables as counts and percentages. Paired comparisons of FVC (% predicted), FEV_1_ (% predicted), and FEV_1_/FVC (%) between the pre- and post-haze assessments were examined using the Wilcoxon signed-rank test, while paired changes in the percentage of participants with FEV_1_/FVC < 70% were analyzed using the McNemar test.

To identify factors associated with changes in FEV_1_/FVC (%), univariate linear regression analyses were first performed for each independent variable (age, sex, smoking status, and health-risk categories). Variables with *p* < 0.20 from the univariate analyses were subsequently included in a multivariate linear regression model to adjust for potential confounders. Regression coefficients (β), 95% confidence intervals (CI), and *p*-values were reported. A two-tailed *p* < 0.05 was considered statistically significant. Given the exploratory nature of the analysis, results from regression models were interpreted cautiously.

## 3. Results

### 3.1. Participant Characteristics

A total of 130 participants were examined over time. The majority were female (75%), with over half (55%) aged 60 or below. A notable fraction (56%) attained education only to primary school or lower, and approximately half (52%) indicated a monthly income of ≤5000 THB. Most participants (75%) were employed in agriculture.

The participants showed low risk categories for diabetes and cardiovascular issues at 45% and 77%, respectively. Most of the participants (95%) were classified as low risk for COPD, with only 5% classified as high risk. Collectively, these attributes (Table 1) describe a predominantly female, agricultural cohort with modest income and generally low chronic disease risk.

### 3.2. Changes in Spirometry Parameters Before and After Haze Period

Median values for spirometry parameters are reported in Table 2. Both FVC (% predicted) and FEV_1_ (% predicted) increased significantly after the haze period (*p* < 0.001 for both), with median FVC rising from 91.34% (IQR: 82.75–97.84) to 97.40% (IQR: 88.83–108.98), and FEV_1_ from 91.87% (85.68–101.35) to 97.20% (89.35–107.23). In contrast, the FEV_1_/FVC ratio showed a modest reduction, from 85.27% (79.12–90.38) to 82.35% (77.00–86.00; *p* = 0.001).

The observed increases in FEV_1_ and FVC may reflect improved familiarity with spirometry procedures during the second assessment. In this context, the reduction in the FEV_1_/FVC ratio should be interpreted cautiously, as it may reflect relative changes between parameters rather than a definitive indication of airway impairment.

### 3.3. Changes in Spirometry Across Health-Risk Groups

Across all risk categories, spirometry parameters generally improved after the haze season, whereas the FEV_1_/FVC ratio showed modest reductions (Table 3). Details of subgroup comparisons by demographic and health-risk characteristics are provided in Supplementary Table S1.

Participants with low to moderate to high DM risk categories showed significant gains in FVC and FEV_1_ (*p* < 0.05), while those with moderate to high DM risk also exhibited a small but significant reduction in FEV_1_/FVC% (86.2 → 81.8%, *p* = 0.019). Among CV-risk groups, FVC and FEV_1_ increased significantly in participants with low and moderate risk, but the FEV_1_/FVC ratio decreased slightly by about three percentage points (*p* < 0.004 and *p* < 0.035, respectively). The high to very high CV-risk group showed no measurable change.

In the COPD-risk analysis, low-risk individuals showed significant increases in FVC and FEV_1_, with a corresponding decline in FEV_1_/FVC% (85.6 → 82.3%, *p* = 0.001). No significant differences were observed in high-risk participants.

Overall, these subgroup patterns indicate variability in spirometry changes across risk categories; however, these findings should be interpreted cautiously, as subgroup analyses were not designed to establish causal relationships.

### 3.4. Changes in Lung Function Category

When lung function was assessed using the FEV_1_/FVC threshold of 70%, a minor change was noted post-haze season. The percentage of participants with normal lung function (≥70%) declined from 96.9% to 93.1%, while those exhibiting lung-function decline (<70%) rose from 2.3% to 6.9%, as detailed in Table 4. Despite the small proportions, the variation was statistically significant (*p* = 0.031). However, when applying the LLN criterion, no statistically significant change was observed between pre- and post-haze assessments (*p* = 0.508).

These findings indicate that the observed changes in lung function categories were sensitive to the classification method used and should be interpreted with caution, particularly given the small number of affected participants.

### 3.5. Factors Associated with Change in FEV_1_/FVC Ratio

Potential predictors of within-subject change in FEV_1_/FVC were examined using univariate linear regression (Supplementary Table S2). Variables with *p* < 0.20 were then entered into a multivariable model (Table 5).

After adjustment, no predictors reached statistical significance. Participants aged ≥60 years, low-income individuals (≤5000 THB), and those with moderate-or-higher DM risk exhibited greater FEV_1_/FVC ratio reductions (β = −2.25, −2.20, and −1.46, respectively; all *p* > 0.05). The model explained a small proportion of variance (R^2^ = 0.061; adjusted R^2^ = 0.038; F = 2.708; *p* = 0.048).

Given the low explanatory power of the model and the absence of statistically significant predictors, these findings should be considered exploratory. No definitive associations between demographic or health-related factors and changes in FEV_1_/FVC can be established based on this analysis.

## 4. Discussion

This study showed a modest reduction in the FEV_1_/FVC ratio post-haze, despite increased lung volumes. Slight FEV_1_ and FVC increases may reflect participant familiarization with spirometry procedures, aligning with prior longitudinal findings on learning effects. In this context, the observed reduction in the FEV_1_/FVC ratio should be interpreted cautiously and does not necessarily indicate definitive airway impairment. This pattern may reflect early functional variation rather than confirmed obstructive change. These findings enhance previous cross-sectional [19] analyses by providing temporal evidence of spirometry variation associated with the haze period in rural Northern Thailand.

### 4.1. Interpretation of Spirometry Changes

The notable post-exposure augmentations in FEV_1_ and FVC likely reflect improved participant proficiency with spirometry procedures rather than true physiological improvement, as indicated by findings in other studies employing repeated measures [22,23]. Conversely, the persistent reduction in FEV_1_/FVC% across various subgroups may reflect relative changes between parameters and should be interpreted with caution. Such changes have been described in populations exposed to particulate matter, although interpretation remains complex due to potential measurement and learning effects. An analogous trend has been documented among urban and occupational populations in Taiwan and Vietnam, wherein recurrent exposure to fine particulate matter was associated with diminished expiratory flow and compromised mid-expiratory function [24,25]. Exposure to fine particulate matter and its associated toxicants promotes oxidative stress–mediated epithelial damage, which may contribute to transient airway responses [20,26].

### 4.2. Comparison with Local and Regional Studies

The observed median reduction in FEV_1_/FVC of approximately three percentage points following haze exposure is consistent with findings from various studies indicating that acute PM_2.5_ exposure is associated with changes in lung function. For instance, Tsai et al. [12] reported significant declines in airway function among children in Taiwan exposed to PM_2.5_, while Guo, C., et al. [27] highlighted long-term impacts on adult lung function trajectories due to particulate exposure. A systematic review indicated that a 10 µg/m^3^ increase in PM_2.5_ correlates with a decrease in peak expiratory flow (PEF) by 1.74 L/min in children, with variations based on asthmatic status and geographic location [28]. Additionally, research on rural populations in Thailand demonstrated detectable spirometry alterations even in low-income cohorts with minimal smoking exposure, reinforcing the notion that haze-related PM_2.5_ can significantly impair lung function [15].

### 4.3. Health-Risk Stratification and Metabolic Associations

Subgroup analyses indicated that individuals with moderate to high DM risk exhibited a notable decline in FEV_1_/FVC%, although these differences were not statistically significant in multivariable analysis. This observation is consistent with previous evidence indicating that long-term exposure to particulate matter, including PM10, is associated with the progression of cardiovascular diseases such as coronary artery disease [29]. This may suggest that individuals with higher cardiometabolic risk could be more susceptible to environmental stressors during haze periods.

Research examining underlying mechanisms indicates that elevated blood glucose levels resulting in systemic inflammation and endothelial dysfunction may exacerbate respiratory reactions to airborne pollutants [30,31]. Previous studies indicate a correlation between diabetes, metabolic syndrome, and compromised pulmonary function, characterized by prevalent restrictive ventilatory patterns and diminished spirometry measurements in affected populations [32,33,34,35]. However, these relationships remain complex and should not be interpreted as causal in the present study.

### 4.4. Mechanistic Considerations

Experimental and biomarker evidence from Chiang Mai and other regions supports the hypothesis that PM_2.5_-induced airway impairment arises from oxidative stress and epithelial barrier disruption. Cao et al. [20] found associations between elevated 8-iso-PGF_2_α and respiratory symptoms during the haze season, while decreased CC_16_ levels were linked to fatigue and poor sleep quality. However, these biomarkers were not measured in the present study and are discussed here for contextual interpretation only. Children exposed to PM_2.5_ experience inflammation in their airways and reduced lung capacity [36]. The epithelial layer in the airway plays a vital role in defense, yet it can be affected by particulate exposure. Additionally, oxidative stress resulting from PM_2.5_ exposure has been linked to increased urinary OH-PAHs [37,38]. These mechanisms provide biological plausibility but should be interpreted cautiously in relation to the present findings.

### 4.5. Public Health and Surveillance Implications

Our findings underscore the utility of repeated community-based spirometry as a low-cost surveillance approach for detecting early functional changes during haze seasons. The modest but consistent reduction in FEV_1_/FVC ratio, even in predominantly nonsmoking agricultural populations, suggests that residents may experience subclinical functional variation. Integrating spirometry with biomarker monitoring and air-quality data could enhance early warning systems and guide interventions such as mask distribution, indoor air filtration, and health-risk communication during peak PM_2.5_ period.

These findings suggest that community-based spirometry may be a practical approach for monitoring functional changes in populations exposed to seasonal air pollution. Evidence from previous studies similarly demonstrates that spirometry can identify early functional variation in populations exposed to biomass-related air pollution [39,40]. Integration with environmental monitoring data may further support early warning and public health response strategies. These findings may also have broader implications beyond respiratory health, as environmental particulate exposure has been linked to systemic cardiovascular effects, including disease progression in coronary arteries [29].

### 4.6. Limitations

This study has several limitations that should be considered when interpreting the findings. The sample size was relatively modest, and individual-level PM_2.5_ exposure was not directly measured, with exposure assessment based on regional ambient monitoring data during the haze season. Therefore, the findings should be interpreted as temporal associations observed during periods of elevated ambient PM_2.5_ rather than direct evidence of individual exposure effects. The cohort was predominantly female and agricultural, which may limit the generalizability of the findings to other populations. Although spirometry was performed by the same certified team following ATS/ERS standards, the observed increases in FVC and FEV_1_ during the second assessment may partly reflect a learning effect, intra-individual variability, or other technical factors related to repeated spirometry measurements. In addition, follow-up was restricted to a single haze season, and seasonal factors such as temperature, humidity, respiratory infections, and changes in physical activity or agricultural practices may also have influenced spirometry outcomes. Future studies should incorporate longer follow-up periods, direct exposure assessment, and larger multi-center populations to strengthen interpretation of the clinical significance of these findings.

## 5. Conclusions

In summary, this longitudinal assessment among Northern Thai residents revealed a small but statistically significant reduction in FEV_1_/FVC% following the haze period, which may be associated with short-term PM_2.5_ exposure. Despite improved spirometry performance, consistent ratio reductions across subgroups were observed. These changes may reflect early functional variation rather than confirmed airway obstruction. These findings highlight the potential value of continued respiratory health monitoring and consideration of periodic spirometry for early detection in high-risk populations in regions affected by recurrent biomass-burning haze.

## Figures and Tables

**Table 1:** Demographic and health risk characteristics of participants (*n* = 130).

Characteristics	Category	n (%)
Sex	Male	32 (25)
	Female	98 (75)
Age group (years)	≤60	72 (55)
	>60	58 (45)
Education	Primary school or lower	73 (56)
	Higher than primary school	57 (44)
Monthly income (THB)	≤5000	67 (52)
	>5000	63 (48)
Occupation	Agricultural worker	98 (75)
	Non-agricultural worker	32 (25)
Diabetes Mellitus (DM) risk	Low	30 (23)
	Low–moderate	29 (22)
	Moderate–high	43 (33)
	High	12 (10)
	Very high	16 (12)
Cardiovascular (CV) risk	Low	100 (77)
	Moderate	25 (19)
	High–very high	5 (4)
COPD risk	Low	123 (95)
	High	7 (5)

Abbreviation: THB, Thai Baht; DM, Diabetes mellitus; CV, Cardiovascular, COPD, Chronic obstructive pulmonary disease.

**Table 2:** Comparison of spirometry parameters before and after haze period.

Parameter	Before Haze Period (Median, IQR)	After Haze Period (Median, IQR)	*p*-Value
FVC (% predicted)	91.34 (82.75–97.84)	97.40 (88.83–108.98)	<0.001 **
FEV_1_ (% predicted)	91.87 (85.68–101.35)	97.20 (89.35–107.23)	<0.001 **
FEV_1_/FVC (%)	85.27 (79.12–90.38)	82.35 (77.00–86.00)	0.001 *
FVC (L)	2.22 (2.00–2.53)	2.39 (2.13–2.80)	<0.001 **
FEV_1_ (L)	1.87 (1.66–2.14)	1.98 (1.75–2.24)	<0.001 **

Abbreviations: FVC, Forced vital capacity; FEV_1_, Forced expiratory volume in one second; IQR, Interquartile range; * *p* < 0.05 and ** *p* < 0.001 by Wilcoxon signed-rank test.

**Table 3:** Changes in spirometry parameters before and after haze period across health-risk groups.

Health-Risk Level	n (%)	FVC (% Predicted) Before → After (*p*-Value)	FEV_1_ (% Predicted) Before → After (*p*-Value)	FEV_1_/FVC (% Ratio) Before → After (*p*-Value)
Diabetes Mellitus (DM) risk				
Low	30 (23)	91.18 → 96.50 (*p* = 0.010) *	91.98 → 97.20 (*p* = 0.010) *	83.70 → 83.55 (*p* = 0.276)
Low–moderate	29 (22)	89.08 → 97.80 (*p* = 0.001) *	89.07 → 94.30 (*p* = 0.009) *	84.49 → 81.50 (*p* = 0.198)
Moderate–high	43 (33)	93.51 → 101.00 (*p* < 0.001) **	101.41 → 102.20 (*p* = 0.030) *	86.23 → 81.80 (*p* = 0.019) *
High	12 (10)	92.05 → 97.60 (*p* = 0.015) *	101.37 → 97.95 (*p* = 0.695)	87.88 → 81.65 (*p* = 0.098)
Very high	16 (12)	88.75 → 95.55 (*p* = 0.010) *	87.75 → 94.45 (*p* = 0.064)	85.50 → 85.30 (*p* = 0.234)
Cardiovascular (CV) risk				
Low	100 (77)	90.93 → 96.80 (*p* < 0.001) **	92.86 → 96.20 (*p* = 0.001) *	85.46 → 82.35 (*p* = 0.004) *
Moderate	25 (19)	95.51 → 99.20 (*p* = 0.012) *	89.69 → 100.70 (*p* = 0.035) *	81.45 → 82.90 (*p* = 0.035) *
High–very high	5 (4)	97.40 → 99.07 (*p* = 0.345)	88.20 → 100.00 (*p* = 0.098)	83.80 → 79.40 (*p* = 0.090)
COPD risk				
Low	123 (95)	91.53 → 98.00 (*p* < 0.001) **	93.59 → 98.50 (*p* < 0.001) **	85.59 → 82.30 (*p* = 0.001) *
High	7 (5)	89.72 → 87.60 (*p* = 0.612)	87.42 → 88.90 (*p* = 0.866)	81.59 → 78.90 (*p* = 0.610)

Abbreviations: FVC, Forced vital capacity; FEV_1,_ Forced expiratory volume in one second; Values are presented as median (% predicted) before and after exposure; * *p* < 0.05 and ** *p* < 0.001 by Wilcoxon signed-rank test. The arrow (→) indicates changes from before to after the haze period.

**Table 4:** Changes in lung function categories before and after haze period.

Classification Method	Lung Function Category	Before Exposure n (%)	After Exposure n (%)	Change (%)	*p*-Value
Fixed ratio (FEV_1_/FVC < 70%)	Normal (≥70%)	126 (96.9)	121 (93.1)	−3.8	0.031 *
	Lung function decline (<70%)	3 (2.3)	9 (6.9)	4.6	
LLN (FEV_1_/FVC z-score < −1.645)	Normal (≥LLN)	122 (93.8)	119 (91.5)	−2.3	0.508
	Obstructive (<LLN)	8 (6.2)	11 (8.4)	2.2	
Total		130 (100)	130 (100)	—	—

Abbreviations: LLN, Lower Limit of Normal; FVC, Forced vital capacity; FEV_1,_ Forced expiratory volume in one second; * *p* < 0.05 by McNemar’s test.

**Table 5:** Multivariable linear regression model for change in FEV_1_/FVC ratio.

Predictor	β (Unstandardized)	95% CI (Lower)	95% CI (Upper)	*p*-Value
Age ≥ 60 years	−2.247	−5.171	0.677	0.131
Low income (≤5000 THB)	−2.198	−5.033	0.636	0.127
DM risk ≥ Moderate	−1.459	−4.310	1.391	0.313

Model summary: R = 0.246; R^2^ = 0.061; Adj. R^2^ = 0.038; F = 2.708; *p* = 0.048. Abbreviations: CI, Confidence interval; THB, Thai Baht; DM, diabetes mellitus.

## Data Availability

The original contributions presented in the study are included in the article; further inquiries can be directed at the corresponding author.

## Supplementary Materials

The following supporting information can be downloaded at: https://www.mdpi.com/article/10.3390/jcm15103791/s1, Table S1 provides subgroup analyses of spirometry parameters before and after haze period across demographic and health-risk categories. Table S2 summarizes univariate linear regression analyses of factors associated with changes in the FEV_1_/FVC ratio.
